# Epidemiology of tuberculosis and susceptibility to antituberculosis drugs in Reunion Island

**DOI:** 10.1186/s12879-022-07965-4

**Published:** 2023-01-05

**Authors:** Moreea Loukman, Belmonte Olivier, Boulay Vincent, Dekkak Rachid, Ferdynus Cyril, Verduyn Morgane, Coolen-Allou Nathalie

**Affiliations:** 1 Service de Pneumologie, Centre Hospitalo-Universitaire Réunion, Allée des topazes CS 11021, 97400 Site St. Denis, France; 2 Service de Microbiologie, Centre Hospitalo-Universitaire Réunion, Site St. Denis, France; 3 Centre de Lutte Anti Tuberculeuse Sud Réunion, St. Denis, France; 4 Centre de Lutte Anti Tuberculeuse Ouest Réunion, St. Denis, France; 5 Methodological Support Unit, Saint-Denis University Hospital, St Denis, France; 6 Clinical Research Department, INSERM, CIC1410, 97410 Saint-Pierre, France; 7 Centre de Lutte Anti Tuberculeuse Nord-Est Réunion, St. Denis, France

**Keywords:** Tuberculosis, Reunion Island, Epidemiology, Resistance, Antituberculosis, Indian Ocean

## Abstract

**Background:**

Tuberculosis is the first fatal infectious agent in the world with 1.2 million annual deaths for 10 million cases. Little is known about the epidemiology of tuberculosis and its resistance in Reunion Island, which is at the heart of migratory flows from highly endemic Indian Ocean territories.

**Methods:**

We carried out a retrospective observational study of cases of tuberculosis disease in Reunion Island between 2014 and 2018. The epidemiological, demographic, microbiological, clinical and social characteristics were analyzed from mandatory declarations, microbiology database and medical files.

**Results:**

265 cases of tuberculosis disease were recorded over the period, ie an incidence of 6.2 / 100,000 inhabitants. 114 patients (43%) were born or resided > 6 months in the rest of the Indian Ocean area. The risk of infection was increased if birth in Madagascar (OR 23.5), Comoros (OR 8.9) or Mayotte (OR 6.8). The prevalence of HIV co-infection was low (2.5%). There were 31 cases (14.4%) of resistance to antituberculosis including 3 (1.4%) of multidrug-resistant tuberculosis and 0 case of extensively drug-resistant tuberculosis. The female gender (61.3% of resistant) was associated with resistance. The resistance rate was not significantly different depending on the geographic origin.

**Conclusion:**

This is the first exhaustive epidemiological study of tuberculosis in Reunion Island. The incidence there is relatively low but increased for people with links to neighboring islands, particularly Madagascar. The prevalence of multidrug resistance is low, with no associated increased risk for patients from the Indian Ocean area.

## Background

Tuberculosis is the first fatal infectious agent in the world with 1.2 million annual deaths for 10 million cases [1]. Tuberculosis eradication remains one of the main goals for World Health Organization (WHO), impeded by limited health care facilities in less developed countries, spreading of HIV pandemic and growing proportion of drug resistant tuberculosis associated with poorer prognosis and frequent therapeutic failure [1, 2]. Spreading of rapid molecular detection of tuberculosis resistance in the past 10 years helped to identify and treat these patients with resistance in low-income countries [3–5].

Reunion Island is a French oversea department situated in the Indian Ocean 10000 km away from metropolitan France, with more than 850,000 inhabitants in 2021. Migratory flows are important with surrounding islands including Mayotte (other French oversea department), Madagascar, Comoros or Mauritius, where incidence of tuberculosis is higher and healthcare resources are more limited. Little is known about epidemiology of tuberculosis in the area. Baroux et al. [6] and recent French Public Health data [7] reported a low incidence in Reunion Island respectively in the 2000–2007 and 2009–2018 periods, but the studies based on mandatory tuberculosis declarations lacked for clinical and microbiological data, including antimicrobial resistance analysis.

Reunion Island is a French oversea department situated in the Indian Ocean 10000 km away from metropolitan France, with more than 850,000 inhabitants in 2021. Migratory flows are important with surrounding islands including Mayotte (other French oversea department), Madagascar, Comoros or Mauritius, where incidence of tuberculosis is higher and healthcare resources are more limited. Little is known about epidemiology of tuberculosis in the area. Baroux et al. [6] and recent French Public Health data [7] reported a low incidence in Reunion Island respectively in the 2000–2007 and 2009–2018 periods, but the studies based on mandatory tuberculosis declarations lacked for clinical and microbiological data, including antimicrobial resistance analysis.

The aim of our study is to determine epidemiology of tuberculosis in Reunion Island and its resistance to antimicrobial treatment, with a specific interest on migratory flows’ impact.

## Methods

The aim of our study was to describe epidemiology of tuberculosis and its resistance in Reunion Island. We conducted a retrospective study including all subjects with tuberculosis from January 2014 to August 2018 in Reunion Island documented by mandatory tuberculosis declaration to the regional health office (Agence Régionale de Santé, ARS) and/or *M. tuberculosis* identification in the microbiological laboratories of the two university hospitals of Reunion Island (St Denis and St Pierre). Tuberculosis cases were defined by microbiological sample positive for *M. tuberculosis*, histological data compatible with tuberculosis (granuloma) and/or medical decision of complete antituberculosis treatment. We could exclude duplicates comparing names, surnames, date of birth and address from the microbiological database and from the nominative reporting of the mandatory declarations. Patients with tuberculosis declaration but no confirmed diagnosis of tuberculosis disease in medical file and patients with tuberculosis declaration for latent tuberculosis were excluded.

We calculated estimated incidence of tuberculosis in Reunion Island population according to their birthplace based on 2013 demographic data from Institut National des Statistiques et des Etudes Economiques (INSEE).

All patients data were recorded from tuberculosis declaration, informatic database of the microbiological laboratories of the two university centers, medical files from the 4 Reunion Island hospitals (St Denis, St Pierre, St Benoit and St Paul) and medical files from the 3 tuberculosis control centers (Centres de Lutte Anti Tuberculeuse, CLAT). We retrospectively reported the following:

- Demographic and epidemiological findings: gender, age, place of birth, place of residence, cumulative length of stays in other country more than 6 months, time from arrival in France
- Social characteristics: health insurance, Directly Observed Therapy, homelessness
- Clinical findings: previous tuberculosis, previous treatment, previous antituberculosis drugs resistance, exposure to patients with MDR tuberculosis, comorbidities, immunosuppressive treatments, organ clinical signs of tuberculosis, delay to diagnosis, hospitalization duration, treatment regimen, treatment duration
- Laboratory findings: number, type, origin and results of mycobacterial samples, phenotypic antibiotic susceptibility testing, genotypic antibiotic susceptibility testing, cyto-pathological findings
- Prognostic findings: one-year outcome on mandatory tuberculosis declaration confirmed by medical file analysis

The isolates of patients were submitted to the local laboratory for smear microscopy, culture on Lowenstein Jensen medium (Biorad Marne la Coquette, France) and BD MGIT liquid medium, (Becton Dickinson, Franklin Lakes, NJ, USA) and identification using the Hain GenoType MTBC reverse line blot (Hain Lifescience, Nehren, Germany). Local laboratory performed for each new tuberculosis cases a genotypic resistance screening for rifampicine (rpoB) and isoniazid (*kat G and inh A*) (Genotype MTBDRplus, Hain Lifescience, Nehren, Germany), and standard phenotypic antibiotic susceptibility testing for isoniazid, rifampicin, ethambutol, pyrazinamide and streptomycin. Since 2016, local laboratory performed rapid molecular antibiotic susceptibility testing on smear-positive samples for *rpoB* resistance genes using GenXpert MTB/RIF (Cepheid). They were referred to the National Reference Center for Mycobacteria in Paris for any other drug susceptibility testing. Multi drug resistant (MDR) tuberculosis and extensively drug resistant (XDR) tuberculosis are defined following World Health Organisation (WHO) guidelines [8]. Primary resistance is defined for one subject as resistance to antituberculosis at first diagnosis of tuberculosis. Secondary resistance is defined as emergence of antituberculosis resistance in one subject treated for tuberculosis.

Results were expressed as total number (percentage) for categorical variables and as median (25–75th percentiles) for continuous variables. Continuous variables were compared using the Mann–Whitney *U* test. Categorical variables were compared using the Chi-square test or the Fisher’s exact test, as appropriate. A *P* value < 0.05 was considered significant. Analyses were performed using Statis and pvalue.io softwares. After bivariate analysis, risk factors for antituberculosis drug resistance were entered into a multivariate logistic regression analysis using backward selection with *P* < 0.15. Collinearity between independent variables was investigated. When identified, the most clinically relevant factor was chosen for use in the multivariate model. A *P* value < 0.05 was considered significant. Analyses were performed using SAS statistical software (8.2, Cary, NC, USA).

Prais-Winsten regression was used to classify the temporal trend of Tuberculosis for each place of birth as increasing, decreasing, or stationary in the study period [9]. The dependent variable was the logarithm base 10 of the rates, and the independent variable was the years of the historical series. Durbin–Watson test was used to assess the existence of a first–order autocorrelation. The percentage of annual variation (APC) and respective 95%CI were calculated [9] as:

$$APC=\left(-1+{10}^{\upbeta }\right)*100.0$$

$$IC95\%=\left(-1+{10}^{{\upbeta }_{min}}\right)*100.0; APC=\left(-1+{10}^{{\upbeta }_{max}}\right)*100.0$$

Every patient received written information about the purpose of the study and could refuse to participate in the study. This study was approved by the Comité d’Evaluation des Protocoles de Recherche Observationnelle of the Société de Pneumologie de Langue Française. Data collection was declared to the Commission Nationale de l’Informatique et des Libertés (French Data Protection Agency or CNIL, MR004).

## Results

**Fig. 1:**
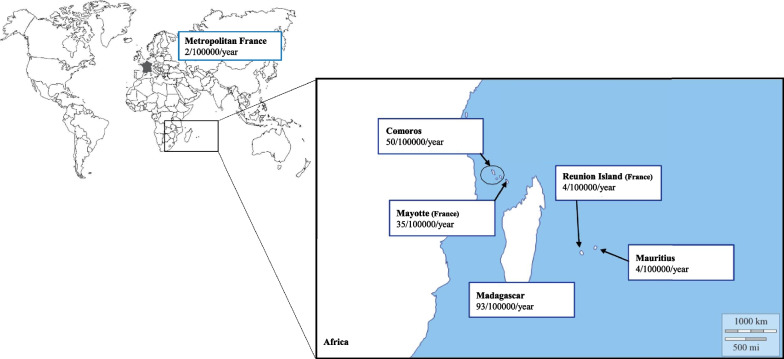
Map of Indian Ocean region: estimated incidence of tuberculosis in Reunion Island patients according to their birthplace

From January 2014 to August 2018, 265 patients were included. Mandatory tuberculosis declaration was recorded in 248 patients (93.6%). The mean calculated annual incidence in Reunion Island was 6.23 per 100,000 inhabitants and was stable over the study period. Using 2013 INSEE data, we estimated incidence according to birthplace as listed in Table 1 and represented on the map in Fig. 1. Incidence was significantly higher in population born in Madagascar, in Mayotte or in Comoros with odd ratios compared to Reunion Island native population of 23.5 [18–30.9], 6.8 [3.7–12.6] and 8.9 [2–4, 4–9, 9–18] respectively.

**Table 1:** Estimated incidence in Reunion Island patients with tuberculosis according to their birthplace

	Total (n = 249)	Population*	Estimated incidence (/100000 per year)	OR**	CI**
Place of birth					
Reunion Island	132 (53)	698 824	4	1	
Madagascar	80 (32.1)	17 179	93	23.5	[18–30.9]
Mayotte	11 (4.4)	6 262	35	6.8	[3.7–12.6]
Metropolitan France	11 (4.4)	92 798	2	0.4	[0.2–0.7]
Comoros	7 (2.8)	2 801	50	8.9	[4.2–18.9]
Mauritius	1 (0.4)	5 097	4	0.62	[0.1–4.4]
Other	5 (2)	na	na	na	na

*Place of birth in Reunion Island population is taken from Institut National des Statistiques et des Etudes Economiques (INSEE) 2013 data. **Odd ratios and confidence intervals are calculated comparing patients born outside Reunion Island to Reunion Island native patients. *OR* odd ratio, *CI* confidence interval, *na* non applicable. Results are expressed in median [25–75th percentiles] or number (percentage)

Over the 2014–2018 period, tuberculosis incidence increased in subjects born in Madagascar as it was stable in patients born in other area (Table 2).

**Table 2:** Evolution of tuberculosis incidence over the 2014–2018 period among Reunion island patients according to their birth place

Place of birth	β (CI 95%) 2004–2018	APC % (CI 95%) 2014–2018	Reading
Reunion Island	0.006 (− 0.06 to 0.07)	1.3 (− 12.8 to 17.8)	Stationary
Madagascar	0.09 (0.03 to 0.14)	22.2 (7.6 to 38.8)	Increasing
Mayotte	− 0.004 (− 0.12 to 0.11)	− 1.0 (− 24.5 to 29.8)	Stationary
Metropolitan France	− 0.03 (− 0.10 to 0.04)	− 6.5 (− 20.3 to 9.6)	Stationary
Comoros	0.00 (− 0.03 to 0.03)	0.0 (− 7.6 to 8.3)	Stationary
Mauritius	NC	NC	NC
Others	NC	NC	NC

β–regression coefficient, 95% CI – confidence interval 95%, *APC* annual percent change

Among our patient cohort, 212 patients had pulmonary tuberculosis in 212 patients (80%), other localizations were ganglionar in 44 patients (16.6%), pleural in 33 patients (12.5%), abdominal in 10 patients (3.8%), osteo-articular in 7 patients (2.6%), pericardic in 5 patients (1.9%), uro-genital in 4 patients (1.5%), cutaneous in 3 patients (1.1%), neurological in 3 patients (1.1%) and laryngeal in 2 patients (0.8%). Microbiological results were available for 228 patients (86%). Respiratory samples were smear positive in 130 patients (53.1%) and culture were positive in 220 patients (83.3%). Cyto-pathological examination showed granuloma with necrotic caseum in 55 patients (20.8%).

Susceptibility testing was available for 216 patients (80.4%). Results are shown in Table 3. Thirty-one patients (14.4%) had anti tuberculosis drug resistance, including 29 patients with primary resistance. Twelve patients (5.6%) had resistance to isoniazid and 3 patients (1.4%) had MDR tuberculosis. There was no XDR tuberculosis. In patients with resistance to streptomycin, treatment was similar to susceptible tuberculosis. In other patients with resistance, fluoroquinolones were used in 8 patients (25.8%), aminoglycosides in 3 patients (9.7%), ethionamide in 2 patients (6.5%), cycloserine in 2 patients (6.5%) and linezolid in 1 patient (3.2%).

**Table 3:** Antituberculosis drugs susceptibility testing in Reunion Island patients with tuberculosis

	n = 216
Anti tuberculosis drugs susceptibility	185 (85.6)
Anti tuberculosis drugs resistance	31 (14.4)
Rifampicin	4 (1.9)
Phenotypic resistance (n = 213)	4 (1.9)
Genotypic resistance: *rpoB* gene mutation (n = 113)	2 (1.8)*
Isoniazid	12 (5.6)
Phenotypic resistance (n = 213) (n = 91)	12 (5.6)
Genotypic resistance (n = 113):	7 (7.7)*
* katG* mutation	4 (4.4)
* inhA* mutation	3 (3.3)
Pyrazinamide phenotypic resistance	14 (6.5)
Ethambutol phenotypic resistance	1 (0.5)
Streptomycin phenotypic resistance	11 (5.1)
Isoniazid monoresistance	4 (1.8)
MDR tuberculosis	3 (1.4)
XDR tuberculosis	0 (0)
Primary resistance	29 (13.2)
Secondary resistance	2 (0.9)

*MDR* Multi Drug Resistant. *XDR* Ultra Drug Resistant*.* Results are expressed in median [25th–75th percentiles] or number (percentage). *All genotypic results were concordant with phenotypic resistance

Clinical and social characteristics are presented in Table 4. Median age was 46 years [32–61]. Five patients (2.5%) were HIV positive. In univariate analysis, patients with susceptible tuberculosis were more likely women, diabetic and had longer treatment. Multivariate analysis showed significantly more women in the resistant group and longer treatment duration (Table 6).

**Table 4:** Clinical and social characteristics in Reunion Island patients with tuberculosis: univariate analysis comparison between patients with or without anti tuberculosis drug resistance

	Total (n = 265)	Susceptibility (n = 185)	Resistance (n = 31)	*p* value*
Age (years)	46 [32–61]	47 [35–61]	45 [36–60]	1
Female gender	112 (32.3)	68 (36.8)	19 (61.3)	0.01
Male gender	153 (67.7)	117 (63.2)	12 (38.7)	0.01
Undernutrition (n = 188)	111 (59)	95 (59.4)	16 (57.1)	0.82
Diabetes mellitus (n = 197)	34 (17.3)	25 (15)	9 (30)	0.045
Chronic ethanolism (n = 212)	26 (12.3)	23 (12.7)	3 (9.7)	0.77
Immunosuppressive treatment (n = 214)	9 (4.2)	8 (4.4)	1 (3.2)	1
HIV (n = 199)	5 (2.5)	5 (2.9)	0 (0)	1
Organ transplant (n = 262)	5 (2.3)	5 (2.7)	0 (0)	1
Dialysis (n = 214)	5 (2.3)	3 (1.6)	2 (6.5)	0.15
Hematologic malignancy (n = 214)	4(1.9)	4 (2.2)	0 (0)	1
Previous tuberculosis (n = 216)	15 (6.9)	11 (5.9)	4 (12.9)	0.24
Hospitalisation duration (days)	20 [8–34]	21 [8–34.5]	16 [7.5–31.5]	0.72
Diagnosis delay (months)	1 [1, 2]	1 [1, 2]	1 [1, 2]	0.65
Treatment duration (months)	6 [6–6]	6 [6–6]	6 [6–9]	0.057
Directly Observed Therapy (n = 165)	63 (38.2)	49 (35.3)	14 (53.8)	0.073
Homelessness (n = 199)	4 (2)	4 (2.4)	0	1
No health insurance (n = 201)	12 (6)	10 (7.1)	2 (7.7)	1

*HIV* Human Immunodeficiency Virus. Results are expressed in median [25–75th percentiles] or number (percentage). *P value between patients with or without anti tuberculosis drug resistance

Demographic data were available in 264 patients including 214 patients with susceptibility testing results (Table 5). Among these 214 patients, 132 (61.7%) patients were born and have always lived in Reunion Island. Among these 132 patients from Reunion Island, 22 patients (16.7%) had resistance to anti-tuberculosis drugs. Among 68 patients who were born or have lived in Madagascar, 7 patients (10.3%) had resistant strains. Among 10patients who were born or have lived in Mayotte, none had resistance. Among 8 patients who were born or have lived in Comoros, 2 patients (25%) had resistant strains. Complete demographic details and their comparison between patients with susceptible or resistance strains are presented in Table 5. Patients who were born or had lived in Madagascar were less likely to have resistance but with no significant differences in multivariate analysis (Table 6).

**Table 5:** Demographic characteristics in Reunion Island patients with tuberculosis: univariate analysis comparison between patients with or without anti tuberculosis drug resistance

	Total	Susceptible	Resistant	p value*
Place of birth	n = 247	n = 172	n = 29	0.12
Reunion Island	132 (53.0)	85 (49.4)	19 (65.5)	
Madagascar	80 (32.1)	59 (34.3)	5 (17.2)	
Mayotte	11 (4.4)	10 (5.8)	0 (0)	
Metropolitan France	11 (4.4)	11 (6.4)	2 (6.9)	
Comoros	7 (2.8)	5 (2.9)	2 (6.9)	
Other	5 (2.0)	2 (1.2)	1 (3.4)	
Mauritius	1 (0.4)	0 (0.0)	0 (0)	
Place of residence	n = 264	n = 183	n = 31	0.08
Reunion Island	231 (87.5)	158 (86.3)	27 (87.1)	
Madagascar	18 (6.8)	15 (8.2)	2 (6.5)	
Mayotte	5 (1.9)	5 (2.7)	0 (0)	
Comoros	5 (1.9)	5 (2.7)	0 (0)	
Other	3 (1.1)	0 (0)	1 (3.2)	
Metropolitan France	2 (0.8)	0 (0)	1 (3.2)	
Mauritius	0 (0)	0 (0)	0 (0)	
Stays abroad > 6 months	n = 187	n = 128	n = 19	0.65
Any country	63 (33.7)	41 (32)	5 (26.3)	
Madagascar	47 (25.1)	30 (23.4)	4 (21.1)	
Mayotte	6 (3.2)	5 (3.9)	0 (0)	
Comoros	4 (2.1)	4 (3.1)	0 (0)	
Other	6 (3.2)	2 (1.6)	1 (5.3)	
No stays abroad	124 (66.3)	87 (68)	14 (73.7)	
Birth, residence or stay > 6 months	n = 264	n = 183	n = 31	
Madagascar	93 (35.2)	68 (37.2)	7 (22.6)	0.12
Mayotte	12 (4.5)	10 (5.5)	0 (0)	0.36
Comoros	8 (3)	6 (3.3)	2 (6.5)	0.32
Time from arrival in Reunion Island (months)	n = 63 30 [8–114]	n = 45 18 [8–12]	n = 7 48 [10–114]	0.81

Results are expressed in median [25–75th percentiles] or number (percentage). *P value between patients with or without anti tuberculosis drug resistance

**Table 6:** Multivariate analysis comparison between Reunion Island tuberculosis patients characteristics with or without anti tuberculosis drug resistance

Variables	Odds ratio (CI 95%)	*p* value*
Male gender	0.225 [0.08–0.64]	0.005
Diabetes mellitus	1.28 [0.37–4.49]	0.69
Treatment duration	0.87 [0.76–1]	0.05
Directly Observed Therapy	1.74 [0.62–4.94]	0.29
Birth, residence or stay > 6 months in Madagascar	0.35 [0.1–1.18]	0.09

*CI* confidence intervals. *P value between patients with or without anti tuberculosis drug resistance

One-year outcome on mandatory declaration showed 180 patients (67.9%) with ended treatment, 1 patient (0.4%) with still on-going treatment, 1 patient (0.4%) with treatment stopped before 4 months, 8 patients (3%) transferred outside Reunion Island, 20 dead patients (7.6%) and 55 patients (20.8%) lost to follow-up.

## Discussion

This is the first exhaustive epidemiological study in Reunion Island. Our strength was to include patients from both mandatory declarations and microbiological database, reducing bias in incidence estimation (6.4% of missing declaration in our study) and giving precise description of demographical, clinical and microbiological patients characteristics, that could not be reported in previous studies uniquely based on declarations [6, 7]. The rate of missing declarations was low. Since 2016, every microbiological result positive for tuberculosis in universitary hospital laboratory is referred to local tuberculosis center to ensure patient treatment, optimize screening around the cases and lower missing declarations.

We confirm low endemicity in Reunion Island with incidence of 6.23 per 100,000 habitants per year in the 2014–2018 period, closed to recent public health data [7] and to national statistics [10]. However, risk is higher for migrant population: 47% of patients were born outside Reunion Island including 32.1% from Madagascar with increasing incidence in this population over the study period. Our estimated incidence in migrant population must be taken with caution as it is extrapolated from 2013 INSEE data but it is coherent with higher incidences in surrounding islands in 2018: 11.5/100000 habitants per year in Mayotte, 13/100000 habitants per year in Mauritius, 35/100000 habitants per year in Comoros and 233/100000 habitants per year in Madagascar [1, 8]. As in other parts of France, the Office Français de l’Immigration et de l’Intégration (OFII) oversees tuberculosis screening in recently arrived migrants, representing 7.9% of tuberculosis cases in France every year [11, 12]. In our study, median delay between arrival in Reunion Island and tuberculosis diagnosis is 30 months. It is corroborant with a maximum risk of tuberculosis in the 4 first years of arrival in United Kingdom in Aldridge et al. cohort [13]. It suggests that chest X-ray should be repeated in the first years of arrival to improve tuberculosis screening on the territory.

Our work is also the first to describe antituberculosis drug resistance in Reunion Island. Previous studies [6, 7] reported declared resistance at diagnosis but without any phenotypic susceptibility testing after culture results and without any rapid genotypic resistance testing before 2016. In our study, antimicrobial susceptibility data were available in 216 out of 265 patients; the first limit was absence of phenotypic testing results in some patients: those with negative cultures and those whose culture was performed in private laboratories; the second limit was absence of rapid genotypic testing results before 2016 in our university laboratory.

Antimicrobial resistance was low in Reunion Island on the study period. MDR tuberculosis represented 1.4% of the tuberculosis cases whereas national reference center and WHO estimate it represents 1.9% of the tuberculosis in France in 2018 and 3 to 5% of the tuberculosis in the world respectively [1, 14]. There was no XDR tuberculosis in our cohort. However, 14.4% of the Reunion Island patients had resistance for at least one first-line antituberculosis drugs, compared to 13.3% in France. Among them, 5.6% had at least isoniazid resistance versus 2.3% in France, and 1.8% had isoniazid monoresistance versus 2.5% in France [14]. This resistance was not associated with a poorer outcome in our patients, but this analysis was limited by small number of patients. Frequent difficulties to treat patients with resistance and/or toxicity with first line antituberculosis drugs suggest developing rapid second-line antituberculosis susceptibility testing [15] in our laboratory to reduce delay for adequate treatment.

We looked for several potential risk factors in antituberculosis drugs resistance.

First, we described mostly primary resistance (29 out of 31 patients). Previous tuberculosis and previous treatment for tuberculosis was higher in patients with resistance than patients with susceptible strains (respectively 12.9% vs. 5.9%) but difference was not significant in these few patients.

Secondly, we considered clinical characteristics. HIV prevalence was low in our cohort. Multivariate analysis showed a higher proportion of women in group with antimicrobial resistance in contrast with previous literature in France or other countries [14]. Besides, diabetes mellitus was more frequent in patients with antituberculosis drugs resistance in univariate analysis, but with no significant differences in multivariate analysis. This corroborates some studies in other specific areas [16–18]. Physiopathological explanations may include delayed time to sputum conversion in diabetic patients leading to secondary resistance, lower rifampicin plasmatic concentrations in diabetic patients and/or chronic inflammation and immunosuppression in diabetic patients making them more susceptible to resistant strains [19]. Since diabetes mellitus is particularly prevalent in Reunion Island [20], it suggests a specific attention to diabetic patients with tuberculosis, who represent 17.3% of our cohort.

Thirdly, we analyzed social characteristics. No significant difference was found when considering health insurance and homelessness between patients with susceptible or resistant tuberculosis. Direct observed therapy was more frequently used in patients with resistance but it did not reach significance. Lost to follow-up was low in our study (20.8% versus 35.2% in France [10], probably due to insularity.

At least, we compared demographic data in patients with or without resistance. Resistance frequency was lower in patients who were born or had lived in Madagascar than patients from Reunion Island. Little is known about antimicrobial resistance in Madagascar, because of extreme poverty and healthcare system difficulties. Between 2012 and 2017, a nationwide program tried to evaluate drug susceptibility in patients with tuberculosis relapse and/or close contacts of MDR tuberculosis. In these high-risk patients, only 4.5% of patients had MDR tuberculosis and none of them had XDR tuberculosis. However, authors estimated that only two thirds of Malagasy patients were diagnosed and that only 1% of them were tested for genotypic drug resistance [21]. Our study is one of the few which systematically tested phenotypic and genotypic anti tuberculosis drug resistance in Malagasy people, reporting a very low frequency of resistance (10.3%). Considering migrants from Comoros or Mayotte islands, there was no available data about tuberculosis resistance in the literature so far. Our work reported no resistance in 10 patients from Mayotte and resistance in 2 out of 8 patients from Comoros but we could not draw any conclusion from these small subgroups. Since Mayotte became a French department in 2009, migratory flows from Comoros islands intensified as well as growing numbers of migrants coming from Central and Austral Africa, with some concerns about potential impact on tuberculosis epidemiology in the area.

## Conclusion

Our work is the first exhaustive study about tuberculosis in Reunion Island. It confirms low endemicity in our island but underlines higher risk in migratory population from neighboring islands, especially in the first years of arrival. Antimicrobial resistance was similar to metropolitan France with no significant higher risk in migrants from Indian Ocean area, but further attention is needed following new migratory flows from African countries.

## Data Availability

The datasets generated and/or analysed during the current study are not publicly available due to privacy and ethical concerns but are available from the corresponding author on reasonable request.

## Abbreviations

ARS: Agence régionale de santé
CLAT: Centre de lutte anti tuberculeuse
INSEE: Institut National des Statistiques et des Etudes Economiques
MDR: Multi drug resistant
XDR: Extremely drug resistant
OR: Odd ratio
CI: Confidence interval
NA: Non applicable
